# Molecular profiling of radical prostatectomy tissue from patients with no sign of progression identifies *ERG* as the strongest independent predictor of recurrence

**DOI:** 10.18632/oncotarget.27294

**Published:** 2019-11-05

**Authors:** Wusheng Yan, Muhammad Jamal, Shyh-Han Tan, Yingjie Song, Denise Young, Yongmei Chen, Shilpa Katta, Kai Ying, Lakshmi Ravindranath, Tarah Woodle, Indu Kohaar, Jennifer Cullen, Jacob Kagan, Sudhir Srivastava, Albert Dobi, David G. McLeod, Inger L. Rosner, Isabell A. Sesterhenn, Alagarsamy Srinivasan, Shiv Srivastava, Gyorgy Petrovics

**Affiliations:** ^1^Henry Jackson Foundation for the Advancement of Military Medicine (HJF), Bethesda, MD, USA; ^2^Center for Prostate Disease Research, Department of Surgery, Uniformed Services University of the Health Sciences and the Walter Reed National Military Medical Center, Bethesda, MD, USA; ^3^John P. Murtha Cancer Center, Walter Reed National Military Medical Center, Bethesda, MD, USA; ^4^Division of Cancer Prevention, National Cancer Institute, NIH, Bethesda, MD, USA; ^5^Joint Pathology Center, Silver Spring, MD, USA; ^*^These authors contributed equally to this work

**Keywords:** prostate cancer, NanoString, prognostic biomarker, biochemical recurrence, ERG

## Abstract

Background: As a major cause of morbidity and mortality among men, prostate cancer is a heterogenous disease, with a vast heterogeneity in the biology of the disease and in clinical outcome. While it often runs an indolent course, local progression or metastasis may eventually develop, even among patients considered “low risk” at diagnosis. Therefore, biomarkers that can discriminate aggressive from indolent disease at an early stage would greatly benefit patients. We hypothesized that tissue specimens from early stage prostate cancers may harbor predictive signatures for disease progression.

Methods: We used a cohort of radical prostatectomy patients with longitudinal follow-up, who had tumors with low grade and stage that revealed no signs of future disease progression at surgery. During the follow-up period, some patients either remained indolent (non-BCR) or progressed to biochemical recurrence (BCR). Total RNA was extracted from tumor, and adjacent normal epithelium of formalin-fixed-paraffin-embedded (FFPE) specimens. Differential gene expression in tumors, and in tumor versus normal tissues between BCR and non-BCR patients were analyzed by NanoString using a customized CodeSet of 151 probes.

Results: After controlling for false discovery rates, we identified a panel of eight genes (*ERG, GGT1, HDAC1, KLK2, MYO6, PLA2G7, BICD1* and *CACNAID*) that distinguished BCR from non-BCR patients. We found a clear association of ERG expression with non-BCR, which was further corroborated by quantitative RT-PCR and immunohistochemistry assays.

Conclusions: Our results identified ERG as the strongest predictor for BCR and showed that potential prognostic prostate cancer biomarkers can be identified from FFPE tumor specimens.

## INTRODUCTION

Prostate cancer affects approximately 1 out of 7 men throughout their life time, with an estimated 174,650 new cases and 31,620 deaths in US in 2019 [1]. Although prostate cancer patients exhibit enormous heterogeneity in terms of disease progression [2], and African American (AA) patients suffer higher incidence and mortality rates than Caucasian American (CA) patients [3], we have limited knowledge of the genes that may contribute to this disparity [4]. The paradigm of prostate cancer diagnosis has, for more than two decades, relied on screening for serum prostate-specific antigen (PSA) and digital rectal examination (DRE) followed by biopsy and confirmatory pathologic analysis [5–7]. Elevated PSA levels can arise from inflammation and enlargement of the prostate, leading to a false positive result [8, 9]. Concerns over overdiagnosis and overtreatment due to the lack of specificity of PSA testing has led changes in the recommendations by US Preventive Services Task Force on PSA screening [10, 11]. Then again, although most localized prostate cancer remain indolent, some tumors continue to progress locally and develop distant metastasis over time [12]. Therefore, there is an urgent need for more specific biomarkers that can detect and distinguish aggressive from indolent disease, and better stratify high-risk cancer early.

Clinical parameters such as Gleason score, tumor stage, margin status, PSA level, extracapsular extension, positive surgical margins, seminal vesicle invasion, and lymph node involvement have been combined to predict prostate cancer outcome with limited success [13]. Attempts to further enhance the predictive power of clinical parameters using tumor-derived gene expression markers have has greatly improved the detection, prognosis, and risk evaluation of prostate cancer [14–19]. For individuals suspected of prostate cancer, several prognostic assays based on mRNA detection of cellular genes using needle-biopsies or radical prostatectomy specimen, such as Prolaris, Oncotype DX^®^ prostate, and Decipher are used to predict poor prostate cancer outcomes and help inform patients on whether repeat biopsies for further evaluation are advisable [14–16, 20, 21]. Despite the progress made in identifying candidate biomarkers, their use in clinical settings have been limited due to the lack of validation through multi-institutional studies and prohibitive cost of these new technologies. Therefore, there remains an urgent need for cost-effective prognostic markers that can predict aggressive disease at an early stage and stratify patients for appropriate treatment options.

We used whole-mounted prostate tissue specimens from a cohort of patients who were treated with radical prostatectomy (RP) and were followed-up for up to ten years. At the time of surgery, these patients had prostate tumors with low grade and stage that revealed no signs of future disease progression but had either progressed to biochemical recurrence (BCR) or showed no sign of progression (non-BCR) during the follow-up period. We hypothesized that prognostic biomarkers for identifying patients that may progress to BCR are present even at the early stage prostate cancer and they could be identified from gene expression profiles of prostatectomy specimens from such a patient cohort. We set out to detect genes differentially expressed in prostate tumor and normal tissue specimens from BCR and non-BCR prostate cancer patients, and through their association with BCR status, to identify genes associated with favorable or adverse pathologic features. RNA expression of the tissues specimen was detected without amplification using the high throughput quantitative profiling NanoString platform [22, 23].

## RESULTS

### RNA isolation and quality control

We isolated RNA from whole-mounted prostate FFPE tissue specimens of a cohort of patients who were treated with radical prostatectomy (Table 1). To ensure the integrity of the purified RNA, we optimized the methods for manual microdissection (Supplementary Figure 1) and RNA isolation (Supplementary Figure 2). RNA samples extracted using our optimized methods for both cell isolation and RNA purification were found to have average RIN of 2.3 (2.0 – 2.6 range). RNA fragments of ≥100 nucleotides, and ≥300 nucleotides represented 86%, and 28% of isolated RNA, respectively (Supplementary Table 1). We used 500 ng of input RNA instead of the recommended input of at least 100 ng of intact total RNA to offset the high level of fragmentation and to achieve the acceptable NanoString readout.

**Table 1:** Clinical characteristics of patients

Characteristics	Prostatectomy discovery cohort (*n* = 63)		
	**BCR (*n* = 21)**	**Non-BCR (*n* = 42)**	***p* value **
Time from RP to BCR1 (median), months	19.4 (2.5–98.6)		
Time from RP to last PSA follow-up, months		97.4 (58.1–166.8)	
Range of surgery year	1998–2008	1997–2007	
Age			
Median (IQR)	58.3 (45–70)	56.4 (40–75)	0.26
< 50	3 (14%)	11 (26%)	
50–60	10 (48%)	17 (41%)	
60–70	7 (33%)	11 (26%)	
> 70	1 (5%)	3 (7%)	
Race			
American Caucasian	14 (67%)	27 (64%)	
American African	6 (28%)	13 (31%)	
Hispanic/Other	1 (5%)	2 (5%)	
Clinical tumor stage			
T1	12 (57%)	24 (57%)	
T2	7 (33%)	17 (40%)	
Unknown	2 (10%)	1 (3%)	
Pre-surgery PSA			
Median (IQR)	4.7 (1.8–13.6)	4.7 (0.7–14.2)	0.74
BMI			
Median (IQR)	27 (19–34)	26 (16–33)	0.11
Prostate weight			
Median (IQR)	39.5 (23.4–48.4)	36.6 (22.3–63.7)	0.46
Signs of further progression			
positive margins	Negative	Negative	
Extra-capsular extension	Negative	Negative	
Seminal vesicle invasion	Negative	Negative	
Pathology GS			
≤ 6 (3+3)	12 (57%)	30 (71%)	
7 (3+4)	9 (43%)	12 (29%)	
Pathology tumor stage			
T2	21 (100%)	40 (95%)	
T3A	0 (0%)	2 (5%)	

BCR = Biochemical recurrence, RP = Radical prostatectomy, IQR = interquartile range, PSA = Prostate-specific antigen, GS = Gleason score.

### Differentially expressed genes in prostatectomy specimens from BCR and non-BCR cases

To identify the genes that are differentially expressed in prostate tumors from patients that progressed to biochemical recurrence and those who did not, we analyzed RNA from all cases using a customized 151-probe CodeSet (Table 2). A total of 135 probe sets of this CodeSet target transcripts from 121 oncogenes, tumor suppressors, and gene fusion variants associated with prostate cancer and cancer in general. These genes were selected based on their association with cancer, specifically prostate cancer, according to the following criteria, supported by at least two publications: (1) have significant differential gene expression in prostate tumor versus normal comparison based on microarray gene expression profiling (data accessible at NCBI GEO database, accession GSE32448 [24]), including *ERG* [25, 26], *ERG8* [27, 28], *ANXA2* [29, 30], *MYO6* [31, 32] and *MAOA* [32, 33]; (2) are regulated by androgen, such as *AMACR* [25, 34, 35], *PSGR* [36, 37], *PCGEM1* [38, 39], [40, 41], and *NKX3.1* [42, 43]; (3) are associated with prognosis of prostate cancer, such as *AR* [44, 45], *EZH2* [46, 47], *C-MYC* [48, 49], *PTEN* [50, 51], and *NCOA2* [52, 53]; (4) are associated with the ETS family of transcription factors detected in GSE32448 [54, 55]; (5) are commonly rearranged in prostate cancer [26, 56, 57]; (6) are involved in prostate cancer cell invasion, such as *SPINK1* [58, 59], *TFF3* [60, 61], *MMP2* and *MMP9* [62, 63]; (7) or are associated with multiple malignancies involving PDGF [64], RAS [65], VEGF [66], EGFR [67], TP53 [65, 68], Interleukin [52], and JAK/STAT signaling pathways [69, 70]. An additional 16 probe sets target five genes that distinguish prostate epithelial from stromal cells [71–74], and 11 house-keeping genes with minimal tumor-normal differential expression identified through gene expression profiling [24] were included as controls.

**Table 2:** NanoString CodeSet of 151 probes for prognostic discovery

Prostate cancer prognosis associated genes	Prostate cancer up-regulated	Prostate cancer down-regulated	Prostate cancer gene fusions	Cancer gene subset	Prostate cancer stroma or epithelial genes
*AK*T1	*AMA*CR	*AMD1*	*(ACSL3)3-(ETV1)6*	*AKT2*	*ALCAM*
*ANXA2*	*BICD1*	*EVA1*	*(C15orf21)2-(ETV1)6*	*BRAF*	*KRT18*
*AR*	*CACNA1D*	*GSTP1*	*(CANT1)1-(ETV4)5*	*CAV1*	*KRT5*
*AURKA*	*CLDN8*	*HOXB13*	*(DDX5)2-ETV4)5*	*EGFR*	*POSTN*
*CAMK2N1*	*CRISP3*	*KLK2*	*(FLJ35294)-(ETV1)5*	*FAS*	*VIM*
*CCND1*	*EPC1*	*KLK3 (PSA)*	*(HERPUD1)1-E4*	*GATA1*	**Housekeeping genes**
*CHD1*	*EPC2*	*LTF*	*(HNRPA2B1)1-(ETV1)2*	*HDAC1*	*ACTB*
*C-MYC*	*ERG (Pan)*	*MSMB*	*(KLK2)1-(ETV4)4*	*HIF1A*	*B2M*
*COL1A1*	*ERG1,2,3*	*NEFH*	*(NDRG1)1-E4*	*HRAS*	*CLTC*
*COL3A1*	*ERG8*	*NKX3.1*	*(SLC45A3)1-(ETV5)8*	*KRAS*	*GAPDH*
*CXCR4*	*ETV1*	*ODC1*	*(SLC45A3)1-E4*	*MMP2*	*GUSB*
*EZH2*	*ETV4*	*ACPP (PAP)*	*T1-(ETV4)2*	*MMP9*	*HPRT1*
*FZD4*	*ETV5*	*PMEPA1*	*T1-E2*	*NOTCH1*	*PGK1*
*HSP27*	*GGT1*	*KLK4 (KLK-L1/Prostase)*	*T1-E3*	*NRAS*	*RPL13A*
*JAG1*	*HOXC6*	*PSCA*	*T1-E4*	*NUMA1*	*RPL27*
*KLF4*	*HPGD*	*STAG1*	*T1-E5*	*PDGFR*	*RPS13*
*MAOA*	*MYO6*	*TMPRSS2*	*T2-E2*	*PIK3CA*	*TUBB*
*MUC1*	*NPY*		*T2-E4*	*RAF1*	
*MYCN*	*PCA3*		*T2-E5*	*STAT1*	
*NCOA2*	*PCGEM1*		*T3-(ETV5)2*	*STAT3*	
*OCT4*	*PLA2G7*		*T3-E4*	*TP53*	
*PARP1*	*PSGR*		*T4-E4*	*VEGFA*	
*PTENP1*	*PSGR2*		*T4-E5*	*VEGFR*	
*PTEN*	*FOLH1(PSMA)*		*T5-E4*	*VEGFR1*	
*SMAD4*	*SPARC*		*T5-E5*	*WNT1*	
*SOX2*	*TMEFF2*		*AGTRAP - BRAF*		
*SPINK1*	*TWIST1*		*SLC45A3 - BRAF*		
*SPP1*			**ETS genes**		
*SPRY1*			*ESE3*		
*SPRY2*			*ETS1*		
*STAG2*			*ETS2*		
*TFF3*			*FLI1*		
*TOP2A*			*SPDEF (PDEF)*		
*ZEB1*					
34	27	17	32	25	16

**Figure 1:**
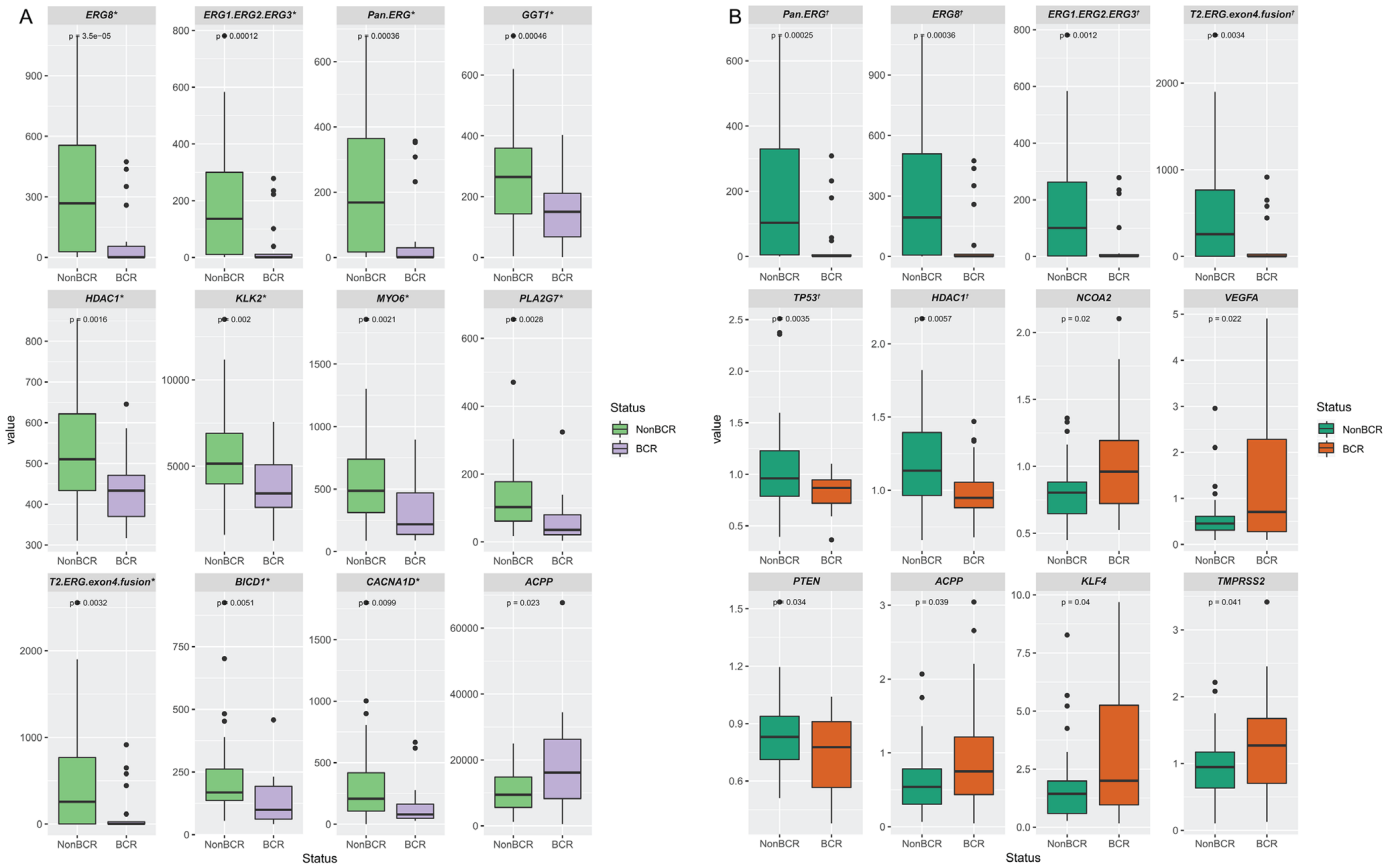
Differentially expressed genes in prostate tissue specimens from patients with BCR or non-BCR detected by NanoString probe sets. Genes that are differentially expressed based on the detection of transcripts in prostate tumors (**A**), and on the ratio of transcripts in tumor vs. normal tissues (**B**). The *p*-value is indicated for each gene. Genes with *q*-value below the cut-off of value of 0.075 and 0.085 for tumor only, and tumor vs. normal analyses are denoted by * and †, respectively, as tabulated in Table 3.

We analyzed both the transcript count in tumor specimens only as well as the ratio of transcript count in tumor compared to normal epithelium. To avoid potential false positives, we used the Storey-Tibshirani method [75] to correct for multiple hypothesis testing. The false discovery rate or *q*-values obtained were used to set the cut-off for selection of markers to avoid the inclusion of false positives or type I errors. By setting the cut-off *q*-value at 0.075 and 0.085 for the evaluation of tumor only, and tumor vs. normal, respectively, we determined that genes with *q*-values below these set values were indeed differentially expressed. Genes that are differentially expressed are shown in Figure 1 and listed together their *p*-values, *q*-values, and expected false positve (FP) values in Table 3. Using this criterion, we identified eight genes, detected by eleven probe sets, that have significantly lower expression in tumors from BCR patients compared to non-BCR patients. In addition to *ERG*, which was detected by *ERG8*, *ERG1/ERG2/ERG3*, *Pan-ERG*, and *T2-ERG-exon4-fusion* probe sets, *GGT1, HDAC1, KLK2, MYO6 PLA2G7, BICD1*, and *CACNAID* were found to have lower expression in tumors of patients that developed BCR. Using the similar criterion, analysis of the ratio of gene expression in tumor compared to normal epithelium identified three genes with significantly different expression profiles between BCR and non-BCR cases (Figure 1B). Specifically, *ERG* isoforms (detected by *Pan-ERG*, *ERG8*, *ERG1/ERG2/ERG3, T2-ERG-exon 4 fusion* probe sets), *TP53* and *HDAC1* were found to have a lower tumor vs. normal ratio in cases that progressed to BCR.

**Table 3:** Differentially expressed genes in prostate tissue specimens of patients with or without progression to BCR based on expression in tumor only and ratio of expression in tumor vs. normal

	**Tumor only**					**Tumor vs Normal**				
	**Probe**	***p* value **	***q*-value **	**Expected FP**	**BCR vs Non-BCR**	**Probe**	***p* value **	***q*-value **	**Expected FP**	**BCR vs Non-BCR** ****
1	*ERG8*	3.50E-05	0.00287	0.003	↓	*Pan.ERG*	0.00025	0.01594	0.016	↓
2	*ERG1.ERG2.ERG3*	0.00012	0.00505	0.010	↓	*ERG8*	0.00036	0.01594	0.032	↓
3	*Pan.ERG*	0.00036	0.00951	0.029	↓	*ERG1.ERG2.ERG3*	0.00124	0.03686	0.111	↓
4	*GGT1*	0.00046	0.00951	0.038	↓	*T2. ERG.exon4.fusion*	0.00342	0.06245	0.250	↓
5	*HDAC1*	0.00158	0.02542	0.127	↓	*TP53*	0.00349	0.06245	0.312	↓
6	*KLK2*	0.00197	0.02542	0.153	↓	*HDAC1*	0.00575	0.08562	0.514	↓
7	*MYO6*	0.00214	0.02542	0.178	↓	*NCOA2*	0.01971	0.24723	1.731	↑
8	*PLA2G7*	0.00283	0.02941	0.235	↓	*VEGFA*	0.02213	0.24723	1.978	↑
9	*T2.ERG.exon4.fusion*	0.00319	0.02947	0.265	↓	*PTEN*	0.03389	0.30341	2.731	↓
10	*BICD1*	0.00509	0.04225	0.422	↓	*ACPP*	0.03911	0.30341	3.034	↑
11	*CACNA1D*	0.00993	0.07502	0.825	↓	*KLF4*	0.04010	0.30341	3.337	↑
12	*ACPP*	0.02298	0.15908	1.909	↑	*TMPRSS2*	0.04074	0.30341	3.641	↑
13	*FAS*	0.03467	0.22155	2.880	↓	*FAS*	0.04674	0.32132	4.177	↓
14	*T2.ERG.exon2.fusion*	0.03828	0.22718	3.180	↓	*HSP27*	0.06458	0.39413	5.518	↑
15	*C.MYC*	0.06511	0.34971	5.246	↓	*T2.ERG.exon2.fusion*	0.06616	0.39413	5.912	↓

### Sensitivity and specificity of ERG probe sets for the detection of BCR

**Figure 2:**
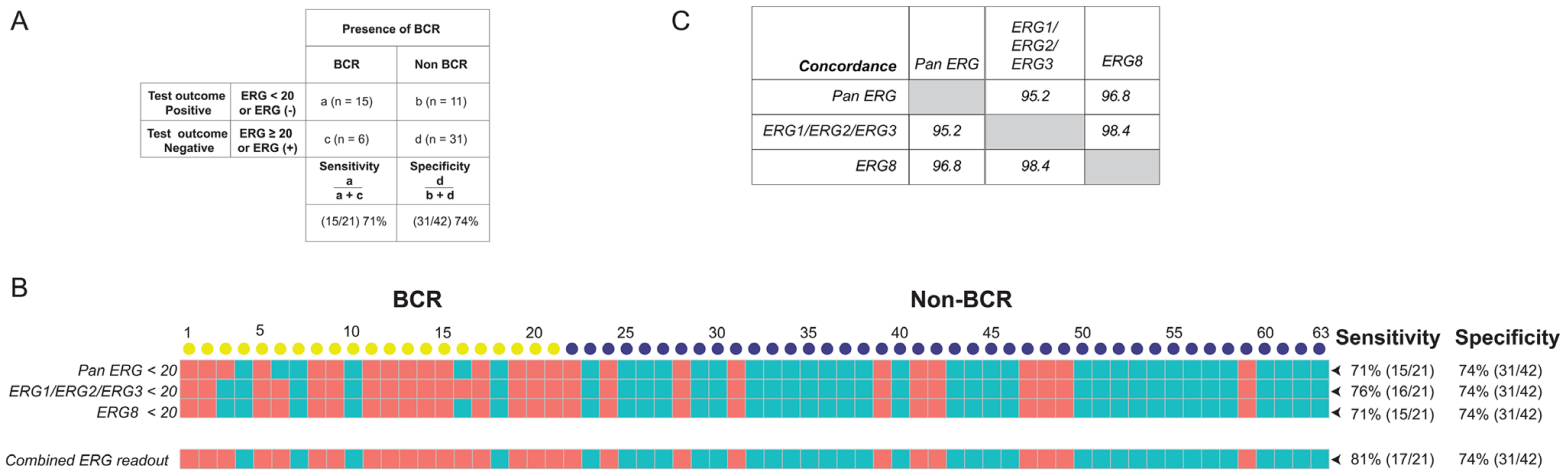
Sensitivity and specificity of ERG specific probe sets and the concordance for predicting BCR. (**A**) Definitions of "sensitivity" and "specificity" are illustrated using transcript counts detected by the Pan ERG probe set. (**B**) ERG status as detected by NanoString probe sets. Transcript counts of <20 were scored as *ERG* negative (represented by salmon colored squares), otherwise as *ERG* positive (represented by teal colored squares). Each column represents an RP specimen: yellow circles represent cases with BCR; blue circles, non-BCR. (**C**) Concordance of *ERG* status between NanoString probe sets targeting *ERG* variants.

To ascertain that the probe sets demonstrate accurately the correlation between gene expression and BCR, the cut-off values were chosen as follows. First, the probe must be significantly different between non-BCR and BCR patients (*p* < 0.05). Second, the probe must show a *q*-value that exclude the likelihood of being a false positive event after adjusting for multiple-hypothesis testing. We then determine the sensitivity and specificity for predicting BCR for each probe set over its range of transcript count and set a cut-off value that prioritize specificity over sensitivity (see Supplementary Figure 3). By setting a cut-off value for detection of transcript counts for each probe set, we convert the continuous numeric transcript counts are into binary values of positive or negative detection. These categorical values allowed us to determine the sensitivity and specificity of not only for individual probe sets, but also for a selected panel of probe sets for predicting BCR. “Sensitivity” or true positive rate measures the proportion of actual positives that are correctly identified as positive, while “specificity” or true negative rate measures the proportion of actual negatives that are correctly identified as negative [76]. Specifically, as illustrated in Figure 2A, by setting the cut-off value of < 20 ERG transcript counts as ERG negative [ERG (-)] to predict a positive outcome for BCR, the sensitivity for BCR of this test is the percentage of cases with BCR that were correctly identified using this criterion as having BCR (15/21 or 71%). By setting the cut-off values of <20 and ≥20 transcript counts to represent ERG negative and ERG positive cases for the probe sets that target *ERG* splice variants or fusion variants, the results showed a strong sensitivity and specificity of these probe sets for predicting BCR. Individually, the *Pan-ERG*, *ERG1/ERG2/ERG3*, and *ERG8* probe sets showed 71%, 76% and 71% sensitivity, respectively, and 74% specificity in predicting BCR (Figure 2B). When used together, these three *ERG* probe sets showed 81% sensitivity and 74% specificity in predicting BCR. The ERG probe sets displayed high concordance of over 95% with one another in their prediction of BCR (Figure 2C).

### Concordance of ERG detection by multiple platforms

**Figure 3:**
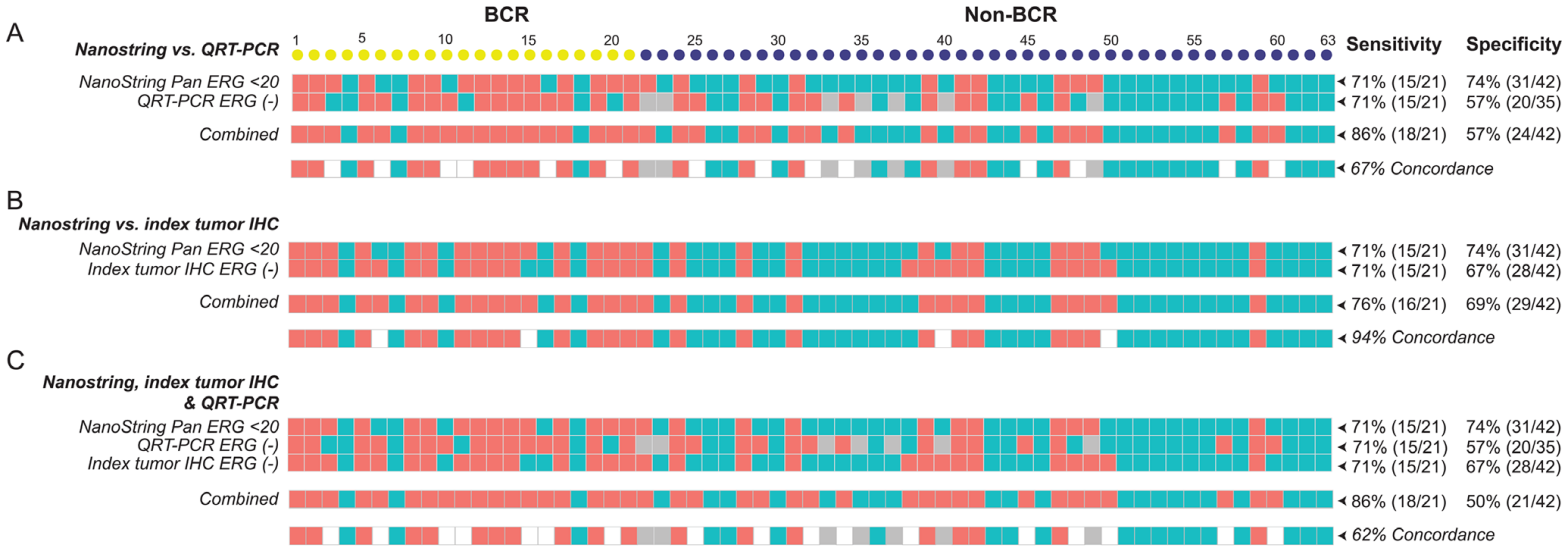
The sensitivity, specificity, and concordance for detecting BCR using NanoString, qRT-PCR, and IHC. The sensitivity and specificity, as well as concordance, for detecting BCR scored by using the NanoString *Pan-ERG* probe set was compared to that scored by qRT-PCR (**A**), and to ERG protein expression of the index tumor, assayed by IHC (**B**), and to results from both qRT-PCR and IHC assays (**C**). In NanoString, cases were scored as ERG negative when transcript count were < 20. In qRT-PCR, Cases with a threshold cycle of 45 or greater for ERG (Ct _ERG_ ≥ 45) were scored as ERG negative and those with a threshold cycle of 38 or greater for GAPDH (Ct _*GAPDH*_ ≥ 38) or had undetectable signals for *GAPDH* were considered as unevaluable (NA). Cases with. *ERG* negative is represented by salmon colored squares; *ERG* positive, teal colored squares; and values that are unavailable, grey colored squares.

Grouping of the detection of *ERG* transcripts into positive and negative categorical values also allowed us to compare the sensitivity of detection of *ERG* mRNA and protein expression using multiple technology platforms. Quantitative RT-PCR (qRT-PCR) amplification of mRNA from the same cohort (*n* = 63) detected 15 *ERG* negative cases among 21 BCR cases, predicting BCR with a sensitivity of 71%, similar to the NanoString Pan-ERG probe set. The assay detected 20 *ERG* positive cases out of 35 evaluable non-BCR cases at a specificity of 57%. When NanoString and by qRT-PCR were used together, the sensitivity and specificity for predicting BCR are 86% and 57%, respectively, achieving a of concordance 67% (Figure 3A). In addition to qRT-PCR, we further compared the detection of ERG transcript by NanoString *Pan-ERG* probe set to the detection of ERG protein expression by IHC assay, an assay routinely used in clinical diagnosis. Although both IHC and NanoString predicted BCR with a sensitivity of 71%, IHC predicted BCR with a specificity of 67%. When used together, the assays achieved a sensitivity of 76%, and a specificity of 69%, reaching a concordance of 94% (Figure 3B). When the NanoString Pan-ERG, qRT-PCR and IHC assays were used in combination, we were able to detect BCR with a sensitivity of 86%, albeit at a reduced specificity of 50%. The concordance among these three platforms for the detection of ERG is 62% (Figure 3C).

### Selection of a gene panel for the prediction of BCR

**Figure 4:**
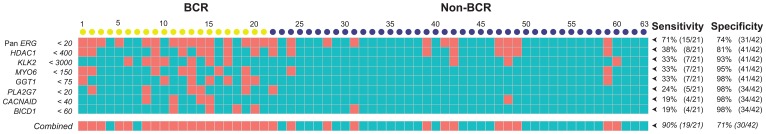
A gene panel for the prediction of BCR was selected based on significant differential expression in prostate tumors of non-BCR and BCR cases. The sensitivity and specificity of a NanoString gene panel consisting of *Pan-ERG, HDAC1, KLK2, MYO6, GGT1, PLA2G7, CACNA1D*, and *BICD1* for BCR; Salmon and teal colored boxes indicate positive and negative scores for each gene, respectively, established based on the cutoff for NanoString transcript counts. Each column represents an RP specimen: yellow circles represent cases with BCR; blue circles, non-BCR. ****

Using our customized NanoString CodeSet, we identified several genes that showed significant differential expression in prostate tumors between non-BCR and BCR cases (*p* < 0.05). As described earlier, the likelihood that these probe sets represent false positive events were excluded by adjusting for multiple-hypothesis testing. The sensitivity and specificity of the probe sets were then determined over the range of their transcript count and a cut-off value that prioritizes specificity over sensitivity is selected for each probe (Supplementary Figure 3). Using this procedure, we were able to establish a panel from individual probe sets that include Pan-*ERG*, *HDAC1*, *KLK2*, *MYO6, GGT1, PLA2G7*, *CACNAID*, and *BICD1* in which the sensitivity of predicting BCR is improved by the addition of each probe set, without subtracting its overall specificity. This combined panel was able to predict BCR with a remarkable sensitivity of 90% and specificity of 71% (Figure 4). Taken together, the analysis of RNA transcripts from FFPE specimens by NanoString identified biomarkers that are differentially expressed during disease progression and therefore, may be useful as prognostic markers of prostate cancer progression.

## DISCUSSION

Biomarkers that differentiate aggressive from indolent prostate cancer could be detected at the level of DNA, RNA (mRNA, miRNA and lncRNA), protein, lipid or metabolite [27, 32, 53, 77, 78]. In this study, we combined the enrichment of homogeneous cancer cells through microdissection and the analysis of RNA without amplification to identify differentially regulated genes in prostate tumors of non-BCR and BCR patients that could be used as prognostic biomarkers for progression to BCR. This is achieved by using a unique CodeSet of probes selected to target cancer, and prostate cancer associated genes or gene fusions on the NanoString nCounter platform. This technology, which can analyze up to 800 genes with digital precision [79], has been used to study gene expression in prostate cancer [23, 80, 81], and other malignancies [82–84].

A significant finding from our study is that the expression level of *ERG*, its variants and *TMPRSS2-ERG* fusion, serve as an independent predictor of BCR in patients with no pathological or clinical signs of progression at surgery. Despite variations in expression of *ERG* detected depending on the probe-set used, likely due to the differential level of splice variants present in tumor tissues, [27, 28], we observed a high concordance of over 95% among the *ERG* probe sets used. This is likely because aberrant *ERG* expression is dependent on the presence of an *TMPRSS2-ERG* fusion event. The detection of *ERG* expression in prostate tumors of non-BCR and BCR cases by NanoString assay was also corroborated by mRNA and protein expression analyses, using qRT-PCR and IHC, respectively. Evidently, the agreement for detecting ERG expression was higher between NanoString and IHC assays (94%) than between NanoString and qRT-PCR (67%). The agreement for detecting ERG expression using all three assays is 62%. These results highlight the limitation of a single biomarker, or platform to detect an event with certainty.

Although both qRT-PCR and NanoString assays detect mRNA the expression, detection of *ERG* transcript by qRT-PCR predicted BCR at a lower specificity (57% vs. 74%). This is likely due to differences in both the quantity of mRNA and the methods used for detecting mRNA expression between these assays: NanoString uses 500 ng of unamplified mRNA and measures transcripts that were bound to probe sets present in excess, while qRT-PCR PCR measures the amplified products from cDNA that were reverse transcribed from 10 ng of mRNA. Furthermore, the smaller amount of input mRNA in qRT-PCR assay may have contributed to the unevaluable cases in qRT-PCR. The lower specificity of IHC for predicting BCR compared to NanoString assay (67% vs. 74%) may arise from RNA splicing or gene fusion events in which the target epitope required for antibody detection in IHC were elided, even though proteins are better preserved compared to RNA in FFPE tissue specimens.

Our results showed that the sensitivity for predicting BCR could be improved when the assays of different modality were used together. Individually, the NanoString, qRT-PCR and IHC assay each predicted BCR with a sensitivity of 71%. When used in pairs, NanoString and qRT-PCR improved the sensitivity for BCR prediction to 86%, while NanoString and IHC assay predicted BCR with 76% sensitivity. Yet, the combined use of all three assays did not improve the sensitivity further, which suggests likely contribution of additional gene alterations to the development of BCR.

Recently, three studies evaluated the prognostic value of selected biomarkers for association with biochemical recurrence as an indicator for prostate cancer progression. Grosset and colleagues examined two tissue microarrays, representing test and validation cohorts, for the association of nuclear NF-kB p65 with BCR, development of bone metastasis and prostate cancer-specific death [85]. They showed, by multivariate analysis, that p65 nuclear localization was an independent predictor for BCR using continuous (Hazard ratio [HR] 1.03; 95% Confidence interval [CI] 1.02–1.04]; *p* < 0.001) and dichotomized (HR 1.60; 95% CI 1.32–1.94; *p* < 0.001) p65 expression data in the validation cohort. In another study, Li and colleagues examined the association between expression of Programmed Cell Death Protein 1 (PD1) and (Programmed Cell Death 1 Ligand 1) PD-L1 proteins with BCR in prostate cancer patients following adjuvant hormonal therapy (AHT) [86]. They reported that overexpression of PDL1 in high risk prostate cancer is significantly correlated with a shorter median time to BCR (*p* = 0.004) after AHT. Univariate analysis identified PDL1-high-expression (*p* < 0.001), and PDL1-high/PD1-negative expression (*p* < 0.001) to be significant risk factors of shorter progression time to BCR in localized disease. PDL1-high-expression was also an independent predictor of time to BCR in multivariate analysis (HR: 3.901; 95% CI: 1.287–11.824; *p* = 0.016). In a third study, Haddad et al. [87] compared the significance of either STAT5 nuclear localization or *STAT5* locus amplification, or both, for predicting BCR after RP. The authors showed that positive status for both events was an independent predictor for shorter disease-free survival, by univariate analysis (*p* < 0.0001), and for BCR, by multivariate analysis (HR = 2.34; *p* = 0.014) after RP.

Several distinct features in our study set it apart from these three studies. Most importantly, the cohort of prostate cancer patients used in this study is ideal for identification of prognostic markers because they had low-risk disease (Gleason score 3+4 or lower) with no signs of future progression at the time of prostatectomy, were followed for many years after surgery and were classified as progressors versus non-progressors based on biochemical recurrence. In addition, the availability of whole mount prostatectomy specimens allowed us to isolate mRNA from tumor and normal cells of each specimen and compare their gene expression. Furthermore, while the racial or ethnic status of the patient cohort were not defined in these recently published studies, our study is based on a diverse patient cohort, consisting of approximately 70% CA and 30% AA patients. Moreover, in evaluating the predictive value of detecting ERG for BCR, our study we not only used the NanoString platform, but we compared it to IHC and qRT-PCR, whereas only IHC [85, 86] or IHC and FISH [87] were used in the other studies. Lastly, we evaluated a total of 121 genes or gene alteration events for association with progression to BCR while the other studies examined the association of either a single biomarker [85], or two interacting proteins [86], or protein expression and copy number amplification associated with a single gene [87].

Our finding on *ERG* and its variants as an independent predictor of BCR in this study is supported by earlier studies from our group and others [88, 89]. Our earlier study had confirmed that ERG expression is more frequent in prostate tumors of CA men in contrast to AA men (49.3% vs. 23.2%) and showed that ERG-negative status in index tumor predicted prostate cancer progression for CA patients by comparing ERG expression in whole-mounted prostate sections from a cohort of 930 patients (336 AA and 594 CA men, [88]. Other studies reported that *TMPRSS2-ERG* fusion or ERG expression are either correlated with progression [90–92] or had no correlation with progression [93–97], after radical prostatectomy. A likely source of this discrepancy is the sampling of prostate tissue specimen examined. Unlike the whole-mounted sections used in this study, which enabled us to assess the multifocal nature of the disease, the other studies examined sections of tissue cores on tissue microarrays [92, 93, 95, 96], biopsies (e.g. trans-rectal ultrasound-guided prostate biopsies) [97] or surgically removed frozen tumors [94], in which tumors with ERG expression or fusion events are likely to be absent or underrepresented. Furthermore, intra-tumor differences in ERG alterations may lead to expression of ERG variants, which could be further exacerbated by inter-tumor heterogeneity [98, 99]. Although fusion of the AR regulated *TMPRSS2* promoter to *ETS related gene* (*ERG*) that results in ERG overexpression [25, 26] is a common event in CA prostate cancer patients (50–70%), it is less frequent among AA and other ethnic groups [100–103]. In these populations, the detection of ERG may no longer be significant as prognostic biomarker for progression to BCR. This emphasizes the need for additional biomarkers that can identify prostate cancer patients with an aggressive disease across populations of diverse ancestries. The detection of ERG expression is, however, especially useful to identify subgroups of patients in which another gene alteration becomes particularly prognostic. For example, in a subgroup of patients with castrate resistant prostate cancer, tumors with ERG-rearrangement but no detectable ERG protein expression may indicate a non-functional AR pathway, suggesting that these patients may not benefit from therapy directed against the AR pathway [104]. Likewise, in subsets of prostate cancer patients with ERG fusion negative, increased expression of PHH3 and Ki-67 [105] or PTEN deletion [106], are associated with increased risk of lethal progression.

Interestingly, evaluation of genes expression ratio between tumor vs. normal tissues of non-BCR and BCR prostate cancer patients identified significantly lower tumor vs. normal ratios for *ERG* variants and *TMPRSS2-ERG* fusion transcripts, as well as for *TP53*, *HDAC1*, and *PTEN* in BCR cases. In contrast, *NCOA2, VEGFA, ACPP, KLF4*, and *TMPRSS2* had significantly higher tumor vs. normal ratio in BCR cases. After controlling for false discovery rates, we conclude that only *HDAC1* and *TP53* have tumor vs. normal ratio between non-BCR and BCR patients that were true positives. Since there were more high confidence genes that were differentially expressed in the tumors between non-BCR and BCR patients, we focused on these genes in our evaluation for a multigene panel.

In addition to the discovery that *ERG* was the strongest predictor for BCR, we detected seven additional genes, *GGT1*, *HDAC1*, *KLK2*, *MYO6*, *PLA2G7*, *BICD1*, and *CACNAID*, which were differentially expressed between non-BCR and BCR prostate cancers and could independently predict BCR in patients without early clinical or pathological signs of progression at surgery. Due to the diverse pathologic features of prostate cancer that range from indolent to metastatic disease, multi-biomarker panels are proven to be more useful for predicting progression than using a single biomarker [14–16, 107]. We evaluated the predictive power of a multi-gene panel for BCR by incorporating the differentially expressed genes that we identified. Together this panel was able to predict BCR with a sensitivity of 90% and specificity of 71%. Further evaluation of these markers using the NanoString assay would benefit from using a larger patient cohort. The assay could be further improved with re-designed CodeSet with probe sets for novel targets, including those that identify tumor heterogeneity, immune response, or actionable gene alterations.

## MATERIALS AND METHODS

### Study design and study subjects

In this retrospective cohort study specimens were collected from RP patients who provided written consent under protocols (#393738, #GT90CM/385525 and #908925) approved by the Institutional Review Boards of the Walter Reed National Military Medical Center (WRNMMC) and the Uniformed Services University of the Health Sciences (USUHS). Specifically, subjects with no BCR were required to have at least 60 months of follow-up (median = 97.4 months); and subjects with BCR, to be event-free for at least 12 months. A BCR event was defined as the detection of at least two consecutive values of serum PSA ≥ 0.2 ng/ml, at ≥8 weeks after RP. Patients who developed BCR were matched to those with no evidence of BCR on both pathologic stage and grade. Patients whose PSA values at diagnosis was ≥20 ng/mL were excluded from both the BCR and non-BCR groups. The clinical characteristics of the patients are shown in Table 1. Of the prostate cancer patients, 21 were classified as individuals with disease progression based on BCR and 42 patients were classified as non-BCR. All cases had a low-grade disease (Gleason pattern 3+3 or 3+4) with no signs of future progression (no positive margins, no extracapsular-extension [ECE] and no seminal vesicle invasion [SVI]). There is no significant difference in other potential prostate cancer recurrence factors such as age, race, pre-surgery PSA level and prostate weight between BCR and non-BCR patients.

### Biospecimen processing and exclusions

Centralized pathology review was conducted by a single genitourinary pathologist at the Joint Pathology Center (formerly the Armed Forces Institute of Pathology) on specimens prepared using a standard whole-mounting technique [99]. Briefly, each prostate was formalin fixed, paraffin embedded, and sectioned at 2.0 µm intervals before mounting whole sections on slides. Each patient specimen was analyzed for Gleason score, perineural involvement, pathologic stage, tumor location, ECE, SVI, tumor volume, and surgical margin status, including the presence of benign glands at the margin. Patients whose pathology review revealed any of the following were excluded from the study: Gleason 8 to10, pT stage 3b to 4, positive surgical margins, nodal involvement, ECE or SVI.

### Improved membrane-frame slide based manual tissue microdissection

Laser capture microdissection (LCM) is routinely used to obtain cells from FFPE tissue sections [108]. To obtain the quantity of RNA sufficient for NanoString analysis, we improved on existing manual microdissection techniques [99] by incorporating a membrane-frame slide to create a “slide-sandwich” (Supplementary Figure 1A). The presence of epithelial cells in histologically defined, matched normal and tumor tissues were first verified by hematoxylin and eosin (H&E) staining. Prior to microdissection, consecutive sections of 7 µm thickness were cut from FFPE tissue blocks in an RNase free environment and mounted onto the “well” side of an RNase AWAY treated poly-ethylene napthalate (PEN) membrane frame slide (ASEE, Cat#DFS-T3-LMD-S-50). After drying, the slides were deparaffinized, stained with Paradise PLUS (developed by Arcturus for staining FFPE tissues), before proceeding with manual microdissection. To avoid introducing potential contamination, the corresponding H&E “guide-slide” is placed onto the “well” side of the membrane-frame dissecting slide. Markings from H&E slide is then transferred to a clean glass slide, which was then placed below the membrane slide to provide support and to guide the dissection. Stromal cells within the targeted area were first removed under 4x objective. Target cells were then excised with a surgical blade by cutting along the periphery enclosing the region of interest and transferred to a new tube (Supplementary Figure 1B).

### RNA isolation and assessment

Total RNA of both tumor and corresponding normal epithelial cells was extracted from micro-dissected FFPE samples using the RNeasy FFPE kit (Qiagen). RNA was separated on the Agilent 2100 Bioanalyzer. RNA quality, as reflected by the RNA Integrity Number (RIN), was evaluated by smear analysis, which assessed the proportion of RNA ≥100 and ≥300 nucleotides (nt) using the Agilent 2100 Expert Software (Supplementary Table 1). Optimization of our RNA isolation steps revealed that RNA quality was better preserved, less fragmented, and gave higher yield when isolated from sections that are freshly recut from archived FFPE blocks, in comparison to RNA that were stored for a week at -80°C following isolation (Supplementary Figure 2A). RNA quality was better preserved by shorter Proteinase K digestion at 56°C of 30 minutes (Supplementary Figure 2B). In a pilot study that compared the yield of RNA isolated from LCM, optimized manual microdissection, and scraping methods were found to have comparable yields (Supplementary Figure 2C).

### NanoString CodeSet design

To identify gene expression associated with prostate cancer outcome, we designed NanoString CodeSet panel consisting of 151 probe sets. The panel was designed to detect 135 target transcripts, which were compiled from 34 genes implicated in or associated with prostate cancer progression, 27 prostate cancer specific gene fusions, 25 cancer associated genes, five genes encoding the *ETS*-family of transcription factors and genes over-expressed (27) or under-expressed (17) in prostate cancer compared to matched benign epithelium. In addition, five prostate stroma or epithelium specific genes were selected as control and 11 housekeeping genes were included for biological normalization (Table 2). Probe sets of 100 bp in length for each gene, which consisted of one capture probe linked to biotin and one reporter probe attached to a color-coded molecular tag, were designed and synthesized at NanoString Technologies (Supplementary Table 2).

### NanoString nCounter analysis

To minimize system derived inter-batch differences, each sample from a BCR patient was assayed together with samples from two non-BCR cases. Hybridization and NanoString nCounter analysis were performed according to the manufacturer’s protocol [82, 109]. Briefly, hybridizations were carried out at 65°C for 20 hours after mixing 5 μL of sample with 10 μL NanoString nCounter reporter probe, 10 μL hybridization buffer and 5 μL capture probe. Hybridization products were then applied to the nCounter Preparation Station for automated removal of excess probe and immobilization of probe-transcript complexes on a streptavidin-coated cartridge. Counts of specific barcodes for individual probe sets were collected using the nCounter Digital Analyzer, and analyzed using the nSolver Analysis Software (Version 2.1.1), available at https://www.nanostring.com/products/analysis-software/nsolver. All 126 samples, (63 tumors and 63 matched benign epithelium), passed quality control metrics for control spike linearity (*R*2 > 0.95) and sensitivity (control spike detection at 0.5 fM). Raw data were normalized against the geometric mean of spiked-in exogenous positive controls to correct the difference resulting from assay efficiency (hybridization, purification, and binding), against the geometric mean of 11 housekeeping genes, which cover a range of constitutive expression levels, to account for variation in the samples, and against spiked-in negative controls to remove hybridization background. All signals below mean background plus two standard deviations were considered as hybridization background and subtracted from the raw data.

### Quantitative RT-PCR (qRT-PCR)

cDNA was reverse transcribed from 10 ng of FFPE derived RNA using gene specific primer pool (GSP) of custom designed reverse primers using Omniscript RT Kit (Qiagen Inc., Germantown, MD) Reverse transcription reactions were performed at 37°C for 60 min followed by 93°C for 5 min, and then held at 10°C. cDNA was preamplified with gene specific primers for *ERG* and Glyceraldehyde 3-phosphate dehydrogenase (*GAPDH*) for eight cycles using the TaqMan^®^ PreAmp Master Mix (Applied Biosystems, CA). Preamplification products were diluted 1:5 and mRNA analysis was performed by TaqMan based qRT-PCR on Stratagene Mx3005P (Agilent Technology, Santa Clara, CA). The forward and reverse primers, and the TaqMan probe for *ERG* are 5′-CAGTATATCCTGAAGCTACGCA AAGA-3′, 5′-GGTCCAGGCTGATCTCCT-3′, and 6FAM-5′-ACTAGGCCAGATTTACCA-3′-TAMRA, respectively. The forward and reverse primers, and the TaqMan probe for *GAPDH*, which was used as internal control are 5′-GAGCCACATCGCCTCAGACACC-3′, 5′-AGAGTTAAAAGCAGCCCTG GTGAC-3′, and [JOE]-ACGACCAAATCCGTTGACTC-TAMRA, respectively. The PCR cycle conditions included an incubation at 55°C for 2 min, a denaturation at 95°C for 10 min, and 50 cycles of 95°C for 30 sec, 56°C for 1 min, and 72°C for 1 min. mRNA expression was analyzed according to relative quantification method, as ΔCt (difference in threshold cycle) = Ct _*GAPDH*_ – Ct _*ERG*_. Fold difference between *GAPDH* control and *ERG* was calculated as 2^ΔCT^ = 2^(ΔCt _*GAPDH*_ − ΔCt _*ERG*_) (Supplementary Table 3). Cases with threshold cycle of 38 or greater for GAPDH (Ct _*GAPDH*_ ≥ 38) or had undetectable signals for *GAPDH* were considered unevaluable (NA). Cases with threshold cycle of 45 or greater for ERG (Ct _ERG_ ≥ 45) were scored as ERG negative.

### Immunohistochemistry assay for ERG

Evaluation of the ERG oncoprotein expression in prostate tissues was performed as previously described [110]. Following deparaffinization, 4 μm sections were dehydrated and blocked in 0.6% hydrogen peroxide in methanol for 20 min. Sections were processed for antigen retrieval in EDTA (pH 9.0) for 30 min in a microwave, followed by 30 min of cooling in EDTA buffer. Sections were then blocked in 1% horse serum for 40 min and incubated with the mouse ERG-MAb (9FY) monoclonal antibody (Biocare Medical Inc., Pacheco, CA) at a dilution of 1:1280 for 60 min at room temperature. Sections were incubated with the biotinylated horse anti-mouse antibody at a dilution of 1:200 for 30 min followed by treatment with the ABC Kit for 30 min, and color was developed by VIP treatment for 5 min using reagents from Vector Laboratories (Burlingame, CA) before they were counterstained by hematoxylin. ERG expression was reported as positive or negative within the specimen. Positive ERG staining of endothelial cells in specimens served as built-in control for the assay.

### Statistical analysis

Normalized raw data were applied for further statistical analysis. NanoString data were analyzed by using the Significance Analysis of Microarray (SAM) tools. Statistical analyses were performed using SAS version 9.3 (SAS Institute, Cary, NC). All *p* values were compared using two-sided statistical tests (summary alpha = 0.05). *P* values for differential expression were used to correct for multiple hypothesis testing using the Storey–Tibshirani method [75] and to identify probe sets that detected statistically significant difference in mRNA expression between Non-BCR and BCR. False discovery rate or *q*-values were computed using the *q value* Bioconductor package [111] in R programming language. In the analyses for differentially expression between Non-BCR and BCR cases in tumor specimens alone, probe sets with *q*-values less than at 0.075 were considered significant. Meanwhile, in the analyses for differential tumor vs. normal ratios between Non-BCR and BCR cases, probe sets with *q*-values less than at 0.085 were considered significant. R 3.6 software [112] was used to present the differential transcript counts as boxplots using the *ggplot2* [113] and *ggpubr* [114] packages.

## CONCLUSIONS

An unmet challenge in prostate cancer is the identification of biomarkers for early detection of aggressive disease. Effective biomarkers with high specificity will provide early treatment options for high risk patients. Using BCR as the endpoint, we analyzed RNA from RP specimens of prostate cancer patients with low Gleason score and show no signs of progression at surgery by using the NanoString platform. *ERG* mRNA expression level, evaluated by NanoString, qRT-PCR, and IHC, was identified as an independent predictor of prostate cancer progression. In addition, seven other genes, *GGT1, HDAC1, KLK2, MYO6, PLA2G7, BICD1*, and *CACNAID*, were found to be differentially expressed in prostate tumors of non-BCR and BCR patients. The tumor vs. normal ratios of *ERG*, *TP53* and *HDAC1* expression in prostate tumors of non-BCR and BCR patients were also found to be significant. Our study, together with publications from other laboratories, highlight the potential of using FFPE tissue as a source for the analysis of prognostic biomarkers for prostate cancer.

## SUPPLEMENTARY MATERIALS





## Abbreviations

BCR: Biochemical recurrence
DRE: digital rectal exam
ECE: extracapsular-extension
FFPE: Formalin-fixed-paraffin embedded
H&E: Hematoxylin and eosin
LCM: laser-capture-micro-dissection
MAb: monoclonal antibody
PEN: poly-ethylene napthalate
RIN: RNA Integrity Number
RP: radical prostatectomy
RT-PCR: real-time polymerase chain reaction
SAM: Significance Analysis of Microarray
SVI: seminal vesicle invasion

## Notes

**Disclaimer**

The contents of this publication are the sole responsibility of the author(s) and do not necessarily reflect the views, opinions or policies of the Henry M. Jackson Foundation for the Advancement of Military Medicine, Inc., the Uniformed Services University of the Health Sciences (USUHS), the Departments of the Army, the Department of Defense (DoD), or any other agency of the U.S. Government. The mention of trade names, specific commercial products, scientific instrumentation, or organizations is considered an integral part of the scientific endeavor and does not constitute endorsement or implied endorsement on the part of the author, DoD, or any component agency.
