# Supplementary material for: Molecular profiling of radical prostatectomy tissue from patients with no sign of progression identifies ERG as the strongest independent predictor of recurrence
Source: Oncotarget. 2019 Nov 5;10(60):6466–83. doi: 10.18632/oncotarget.27294 (PMC6849651; doi:10.18632/oncotarget.27294)
Supplement: Supplementary file 1 [file oncotarget-10-6466-s001.pdf]

Molecular profiling of radical prostatectomy tissue from patients with no sign of progression identifies *ERG* as the strongest independent predictor of recurrence

SUPPLEMENTARY MATERIALS

Isolation and quality control of RNA from prostatectomy specimens

To achieve the desired RNA yield and quality, we optimized tissue section storage and RNA isolation steps (Supplementary Figure 1) We noted RNA isolated from tissues sections that were freshly cut from archived FFPE blocks was better preserved than RNA isolated from FFPE sections and stored for a week at –80°C (Supplementary Figure 2A). Shorter Proteinase K digestion at 56°C resulted in less RNA fragmentation (Supplementary Figure 2B). Based on these results, we conducted a pilot study using five FFPE prostate cancer specimens using freshly cut tissue sections and 30 minutes of Proteinase K digestion to verify if optimal RNA yield could be achieved. The results showed that the RNA yield from modified manual microdissection and from scraping were comparable (Supplementary Figure 2C). RNA isolated from benign epithelium by LCM were more fragmented likely due to the prolonged dissection at room temperature. We obtained approximately 500 ng of total RNA from about 0.6 cm^2 of tumor area of a 7 µm FFPE tissue section and detected 93% target genes. As a quality control, we checked readout of the prostate epithelium specific genes by NanoString. Both tumor and normal cells from RP specimens showed comparable levels of expression of prostate epithelial marker genes such as *KLK3*, *MSMB* (Microseminoprotein Beta) and *ACPP* (Prostatic Acid Phosphatase) (Supplementary Figure 1C). Likewise, prostate cancer genes such as *PSGR*, *AMACR* and *PCA3* were up-regulated in tumor samples. Taken together, the results indicated that our manual microdissection method, improved by incorporating a membrane-frame slide, enabled the molecular profiling of archived FFPE specimen by NanoString.

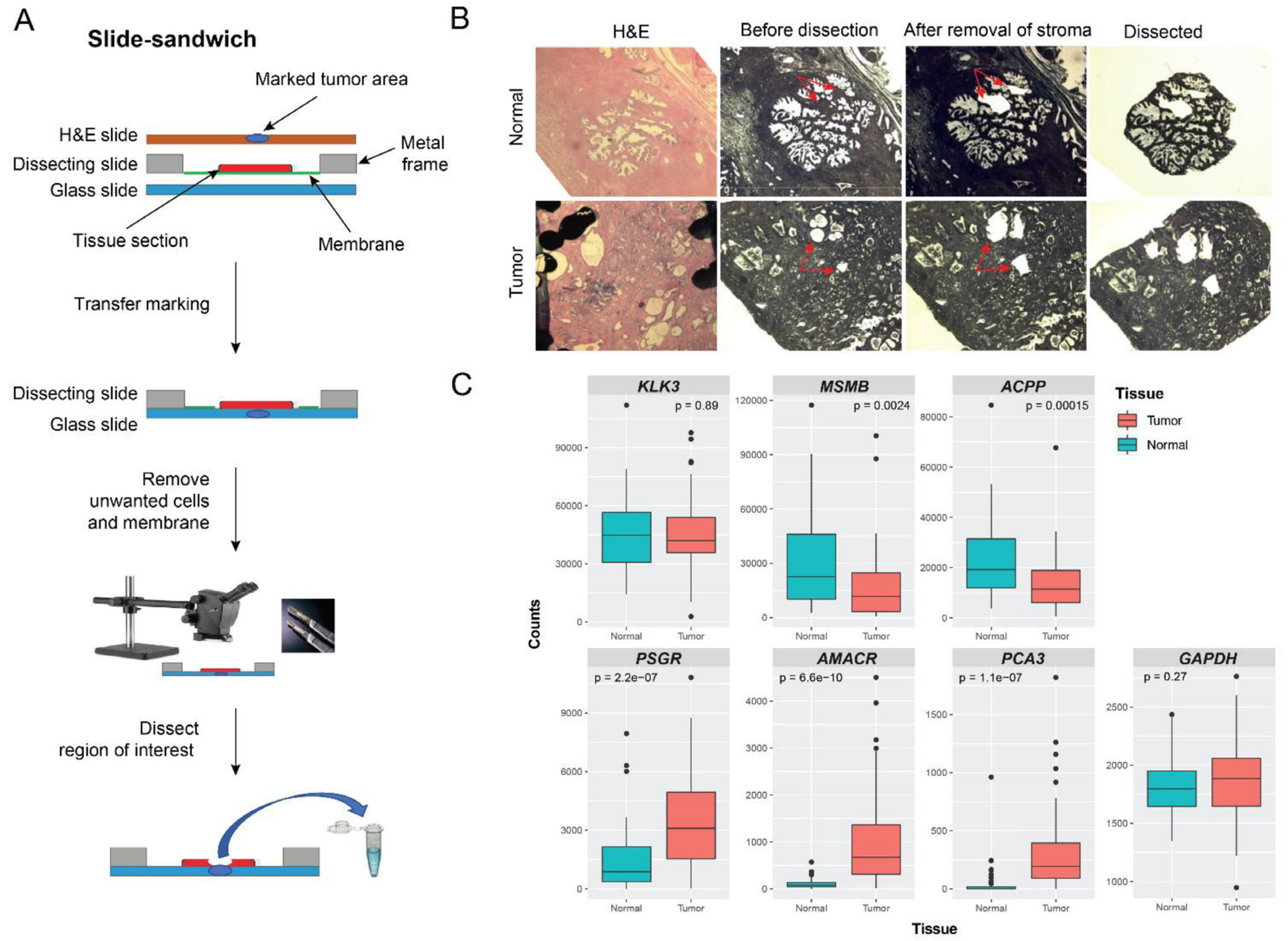

Supplementary Figure 1: Experimental workflow for the manual microdissection of tumor and normal cells from prostatectomy specimens. (**A**) Schematic representation of the steps involved in the membrane-frame slide based manual microdissection. A "slide-sandwich" was made by placing the H&E "guide-slide" on the "well" side of the dissecting membrane slide, and a clean glass slide below to provide support. After matching the tissue outline between the dissecting and guide slides, the markings of tumor area labeled by the pathologist in the top H&E "guide-slide" were transferred to the support glass slide; (**B**) Under a stereo microscope, unwanted cells within the marked area were first excised using a scalpel blade along with the underneath membrane. The selected areas were then manually dissected and collected in labeled microcentrifuge tubes for RNA extraction; (**C**) Expression of prostate epithelial genes (*KLK3*, *MSMB* and *ACPP*), prostate cancer specific genes (*PSGR*, *AMACR* and *PCA3*), and control *GAPDH* gene. T, tumor; N, matched benign epithelium.

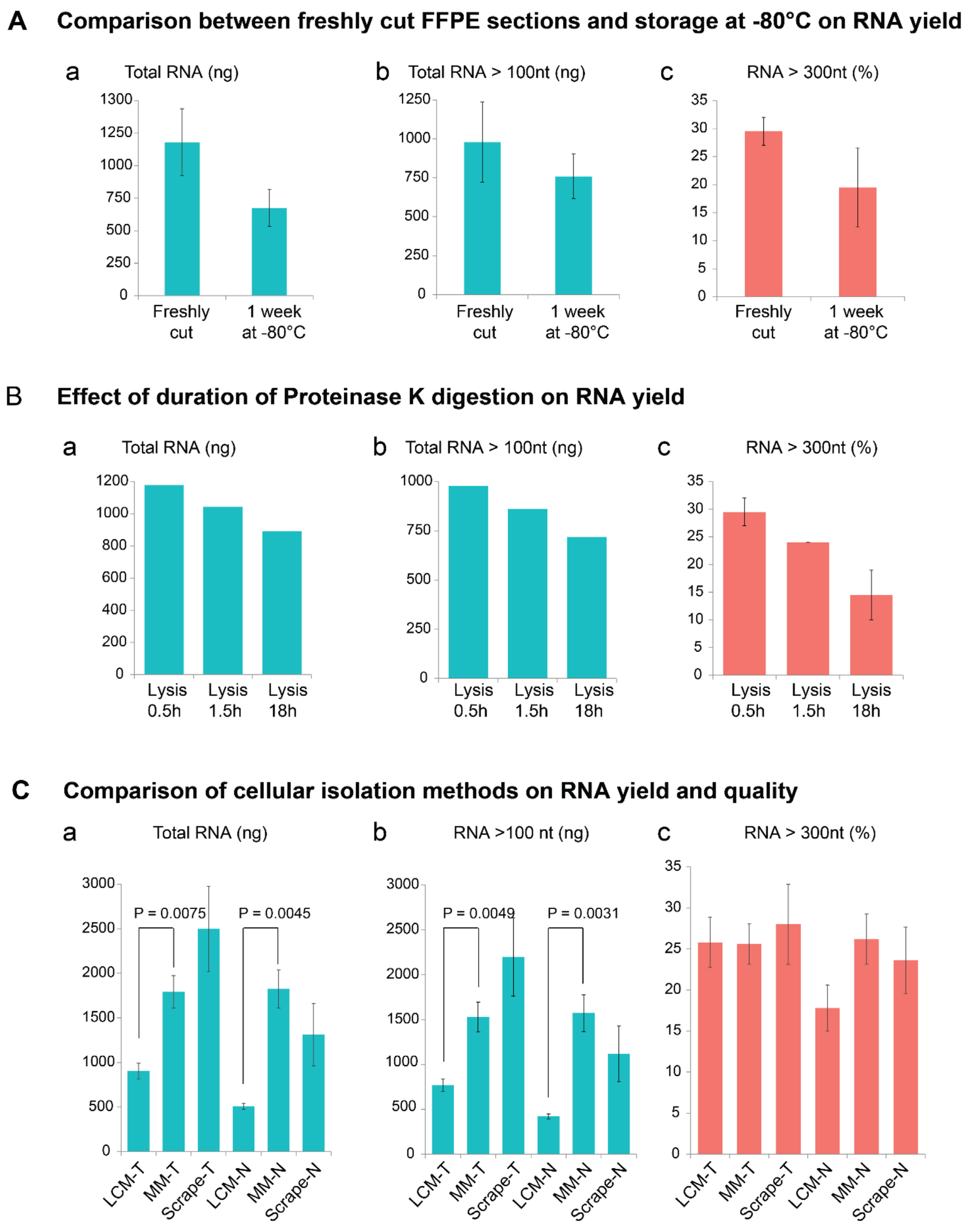

Supplementary Figure 2: (**A**) Comparison between freshly cut FFPE tissue specimens and storage of cut sections for a week at –80°C on yield and quality of total RNA (**Aa**), RNA > 100 nt (**Ab**) and RNA > 300 nt (**Ac**). (**B**) Effect of lysis conditions on the yield and quality of total RNA (**Ba**), RNA > 100 nt (**Bb**) and RNA > 300 nt (**Bc**). (**C**) Comparison of LCM, manual microdissection (MM) and scraping (Scrape) methods on the yield and quality of total RNA (**Ca**), RNA > 100 nt (**Cb**) and RNA > 300 nt (**Cc**) isolated from tumor and normal FFPE tissue specimen.

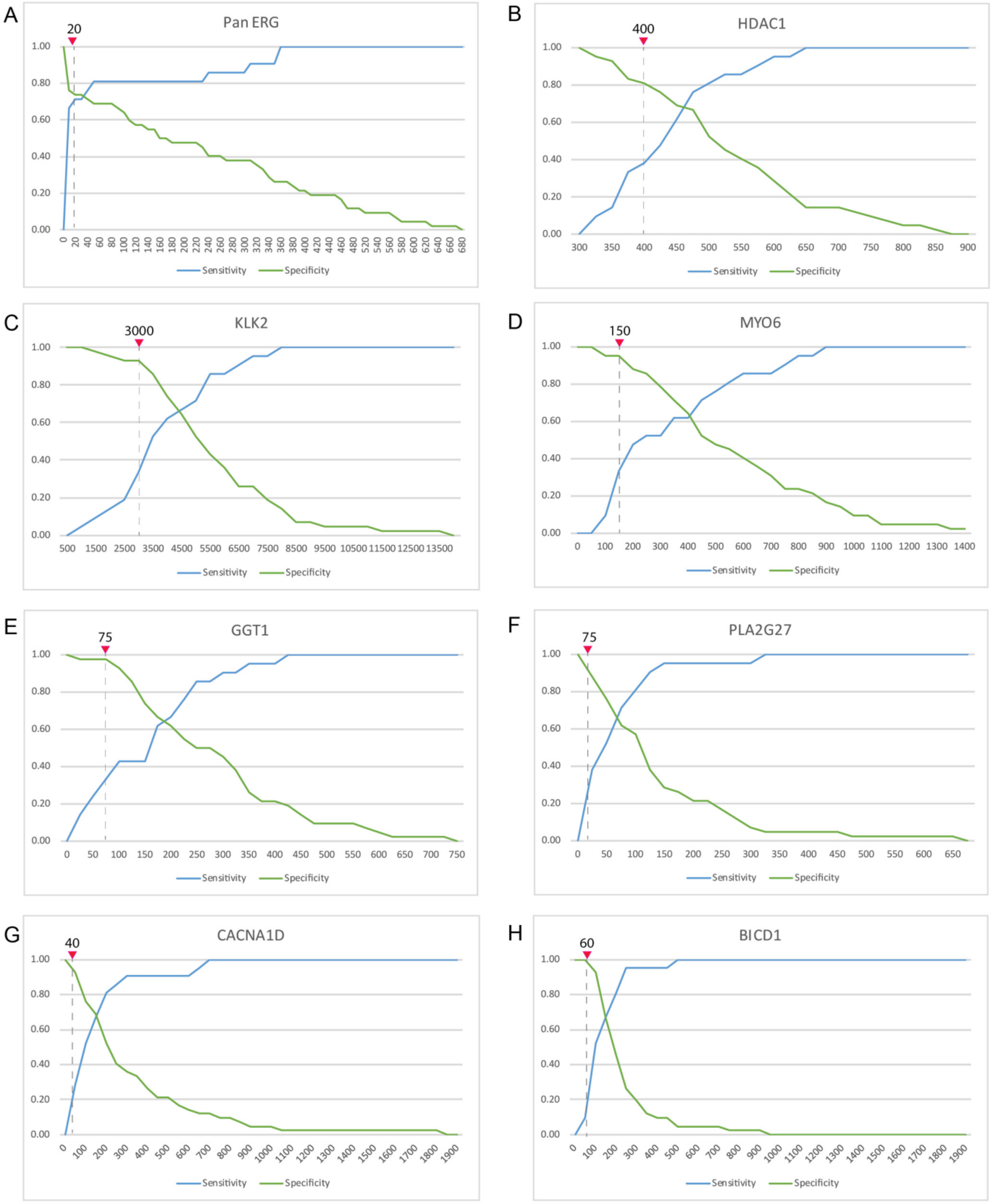

Supplementary Figure 3: Plot of the sensitivity and specificity of genes based on their range of transcript count detected by NanoString nCounter. Sensitivity and specificity plots for Pan-ERG (**A**), HDAC1 (**B**), KLK2 (**C**), MYO6 (**D**), GGT1 (**E**), PLA2G27 (**F**), CACNA1D (**G**), and BICD1 (**H**), showing the cut-off value for each gene that was selected to prioritize specificity over sensitivity.

Supplementary Table 1: BioAnalyzer analysis of RNA samples prepared for NanoString from micro-dissected index tumor and benign epithelium cells, assessed for percentage of RNA >100 and >300 nucleotides

Case no.	Cell population	>100 nt (%)	>300 nt (%)	RIN (%)	Cell population	>100 nt (%)	>300 nt (%)	RIN (%)	BCR status
1	Tumor	86	26	2.3	Normal	85	28	2.2	BCR
2	Tumor	83	20	2.3	Normal	85	31	2.3	Non-BCR
3	Tumor	76	14	2.4	Normal	67	6	2.6	Non-BCR
4	Tumor	83	20	2.3	Normal	85	22	2.3	BCR
5	Tumor	86	27	2.3	Normal	87	28	2.3	Non-BCR
6	Tumor	85	21	2.3	Normal	85	24	2.3	Non-BCR
7	Tumor	84	27	2.3	Normal	81	22	2.3	BCR
8	Tumor	86	30	2.3	Normal	90	27	2.2	Non-BCR
9	Tumor	82	25	2.3	Normal	85	26	2.3	Non-BCR
10	Tumor	85	24	2.2	Normal	90	29	2.1	BCR
11	Tumor	82	25	2.3	Normal	84	24	2.3	Non-BCR
12	Tumor	80	20	2.3	Normal	82	20	2.3	Non-BCR
13	Tumor	86	19	2.4	Normal	87	20	2.3	BCR
14	Tumor	82	23	2.4	Normal	83	22	2.3	Non-BCR
15	Tumor	83	21	2.4	Normal	82	22	2.3	Non-BCR
16	Tumor	89	38	2.1	Normal	91	39	2.1	BCR
17	Tumor	87	26	2.2	Normal	87	26	2.3	Non-BCR
18	Tumor	82	20	2.4	Normal	90	33	2.1	Non-BCR
19	Tumor	89	33	2.1	Normal	89	32	2.2	BCR
20	Tumor	79	33	2.3	Normal	80	19	2.4	Non-BCR
21	Tumor	80	16	2.4	Normal	81	18	2.4	Non-BCR
22	Tumor	88	31	2.2	Normal	89	28	2.2	BCR
23	Tumor	89	40	2.3	Normal	90	38	2.2	Non-BCR
24	Tumor	81	26	2.3	Normal	85	28	2.2	Non-BCR
25	Tumor	88	38	2.2	Normal	90	37	2.2	BCR
26	Tumor	76	17	2.3	Normal	81	14	2.4	Non-BCR
27	Tumor	85	27	2.3	Normal	88	33	2.2	Non-BCR
28	Tumor	87	35	2.3	Normal	92	43	2	BCR
29	Tumor	77	16	2.4	Normal	81	21	2.3	Non-BCR
30	Tumor	85	22	2.3	Normal	82	28	2.3	Non-BCR
31	Tumor	87	34	2.4	Normal	89	27	2.4	BCR
32	Tumor	84	26	2.4	Normal	88	33	2.3	Non-BCR
33	Tumor	86	27	2.2	Normal	92	35	2.2	Non-BCR
34	Tumor	91	35	2.2	Normal	90	27	2.4	BCR
35	Tumor	89	29	2.2	Normal	96	41	2	Non-BCR
36	Tumor	92	40	2.1	Normal	88	37	2.1	Non-BCR
37	Tumor	88	38	2.1	Normal	92	44	2.2	BCR
38	Tumor	88	35	2.2	Normal	89	38	2.2	Non-BCR
39	Tumor	89	35	2.3	Normal	83	36	2.3	Non-BCR
40	Tumor	92	43	2.1	Normal	91	40	2.2	BCR
41	Tumor	92	42	2.1	Normal	93	42	2.1	Non-BCR
42	Tumor	87	33	2.3	Normal	89	25	2.3	Non-BCR
43	Tumor	91	35	2.2	Normal	92	33	2.2	BCR
44	Tumor	86	29	2.3	Normal	87	31	2.2	Non-BCR
45	Tumor	89	32	2.2	Normal	88	31	2.3	Non-BCR
46	Tumor	92	32	2.2	Normal	89	34	2.2	BCR

47	Tumor	89	30	2.4	Normal	92	28	2.4	Non-BCR
48	Tumor	88	32	2.4	Normal	91	40	2.3	Non-BCR
49	Tumor	81	29	2.2	Normal	91	26	2.3	BCR
50	Tumor	90	33	2.2	Normal	70	34	2.4	Non-BCR
51	Tumor	87	30	2.4	Normal	85	20	2.4	Non-BCR
52	Tumor	76	14	2.4	Normal	80	17	2.4	BCR
53	Tumor	72	5	2.6	Normal	78	17	2.4	Non-BCR
54	Tumor	82	22	2.3	Normal	73	11	2.5	Non-BCR
55	Tumor	75	14	2.5	Normal	75	12	2.4	BCR
56	Tumor	84	22	2.3	Normal	83	25	2.3	Non-BCR
57	Tumor	87	19	2.3	Normal	89	21	2.3	Non-BCR
58	Tumor	89	29	2.2	Normal	91	29	2.2	BCR
59	Tumor	82	15	2.4	Normal	80	13	2.4	Non-BCR
60	Tumor	86	14	2.2	Normal	89	31	2.2	Non-BCR
61	Tumor	87	33	2.2	Normal	86	29	2.4	BCR
62	Tumor	90	29	2.3	Normal	76	46	2.3	Non-BCR
63	Tumor	87	29	2.4	Normal	89	39	2.3	Non-BCR
	Average	85.2	27.0	2.3	Average	85.8	28.3	2.3	

Supplementary Table 2: Sequence-specific probes designed for NanoString assay. See Supplementary Table 2

Supplementary Table 3: Comparison of ERG detection by NanoString, IHC and QRT-PCR methods in prostatectomy specimens

			NanoString		IHC		QRT-PCR	
**No**	**BCR**	**Pan ERG**	**ERG1/ ERG2/ERG3**	**ERG8**	**Index tumor**	**GAPDH (Ct_1)**	**ERG (Ct_2)**	**ERG(+)**
1	BCR	0	0	0	0	30.9	No Ct	0
2	BCR	0	0	0	0	31.1	No Ct	0
3	BCR	17	39	77	0	34.4	44	1
4	BCR	308	223	351	1	27	34.1	1
5	BCR	0	0	0	0	30.8	45	0
6	BCR	30	7	12	0	32.1	No Ct	0
7	BCR	356	235	436	1	29.1	37.1	1
8	BCR	5	0	0	0	30.6	No Ct	0
9	BCR	0	0	0	0	28.2	No Ct	0
10	BCR	352	278	474	1	27.7	No Ct	0
11	BCR	0	0	0	0	36.5	32.6	1
12	BCR	0	0	0	0	28.7	No Ct	0
13	BCR	0	0	0	0	28.4	No Ct	0
14	BCR	0	0	0	0	29.9	No Ct	0
15	BCR	0	0	14	1	34.1	47.3	0
16	BCR	48	11	55	1	27.8	No Ct	0
17	BCR	0	0	0	0	33.9	47.4	0
18	BCR	232	102	258	1	32.3	31.8	1
19	BCR	0	0	0	0	31	No Ct	0
20	BCR	2	0	0	0	29.1	43.7	1
21	BCR	0	0	6	0	30.3	No Ct	0
22	Non-BCR	11	0	9	0	NA	NA	NA
23	Non-BCR	268	303	519	1	NA	NA	NA
24	Non-BCR	0	0	0	0	23	No Ct	0
25	Non-BCR	234	346	567	1	33.3	46.4	0
26	Non-BCR	118	51	181	1	31.3	38.1	1
27	Non-BCR	139	103	239	1	31.3	43	1
28	Non-BCR	0	0	0	0	31.6	No Ct	0
29	Non-BCR	91	105	167	1	34.7	No Ct	0
30	Non-BCR	340	325	683	1	32.5	42.1	1
31	Non-BCR	0	0	9	0	28.3	No Ct	0
32	Non-BCR	158	203	421	1	31	No Ct	0
33	Non-BCR	50	68	81	1	40.1	No Ct	NA
34	Non-BCR	622	408	830	1	31.5	No Ct	0
35	Non-BCR	155	248	470	1	NA	NA	NA

36	Non-BCR	331	159	322	1	27	30.4	1
37	Non-BCR	566	412	774	1	NA	NA	NA
38	Non-BCR	580	781	1098	0	34.5	37.6	1
39	Non-BCR	0	0	0	0	26.3	No Ct	0
40	Non-BCR	33	74	102	0	NA	NA	NA
41	Non-BCR	0	0	0	0	30.5	45.8	0
42	Non-BCR	4	0	0	0	34.2	No Ct	0
43	Non-BCR	338	208	401	1	34	41.9	1
44	Non-BCR	403	329	380	1	33.7	35.5	1
45	Non-BCR	179	85	213	1	34.9	No Ct	0
46	Non-BCR	463	216	653	1	27.4	33.1	1
47	Non-BCR	0	0	0	0	31.1	No Ct	0
48	Non-BCR	0	0	0	0	32.1	38.4	1
49	Non-BCR	0	0	0	0	NA	NA	NA
50	Non-BCR	90	130	194	0	30.5	40.4	1
51	Non-BCR	313	238	477	1	29.6	30	1
52	Non-BCR	452	583	812	1	37.9	30.3	1
53	Non-BCR	679	355	970	1	25.3	31.6	1
54	Non-BCR	373	268	630	1	30.4	38.5	1
55	Non-BCR	326	143	348	1	31.5	28.2	1
56	Non-BCR	503	290	479	1	29.8	38	1
57	Non-BCR	104	40	205	1	30.8	No Ct	0
58	Non-BCR	237	130	246	1	28.7	36.7	1
59	Non-BCR	0	0	0	0	28.6	No Ct	0
60	Non-BCR	229	146	290	1	30.7	49.1	0
61	Non-BCR	463	387	771	1	29	33.9	1
62	Non-BCR	103	99	169	1	31.7	44.4	1
63	Non-BCR	383	351	574	1	30.6	40.1	1
